# Rapid differentiation of viable and inactivated African swine fever virus by a viability quantitative PCR

**DOI:** 10.1186/s13567-025-01641-6

**Published:** 2025-10-28

**Authors:** Gaocheng Fan, Xiaowei Gao, Hang Xu, Xinying Dong, Hao Song, Yanhui Fu, Jing Li, Yuying Yang, Hua-Ji Qiu, Yuzi Luo

**Affiliations:** 1 State Key Laboratory of Animal Disease Control and Prevention, Harbin Veterinary Research Institute, Chinese Academy of Agricultural Sciences, 678 Haping Road, Harbin, 150069 China; 2 College of Animal Science, Yangtze University, Jingzhou, 434023 Hubei China

**Keywords:** African swine fever virus, viability quantitative PCR, viral presence, pathogen inactivation, PMA, EMA

## Abstract

Real-time quantitative PCR (qPCR) has been widely used for African swine fever virus (ASFV) surveillance, but it cannot differentiate viable from inactivated virions, as inactivated particles often retain intact nucleic acids. This limitation may lead to overestimation of the presence of viruses in tested samples, potentially resulting in unnecessary outbreak responses and increased economic burdens. To address this challenge, a viability qPCR (V-qPCR) assay was established, utilizing nucleic acid intercalating dyes—propidium monoazide (PMA) or ethidium monoazide (EMA), to discriminate viable and inactivated ASFV. These dyes selectively penetrate compromised viral envelopes in inactivated particles, covalently binding viral DNA, and inhibiting PCR amplification. The optimized V-qPCR assay demonstrated a detection limit of 10^1.5^ TCID_50_/mL for infectious ASFV and effectively discriminated chemically inactivated virus (10^4.5^ TCID_50_/mL) treated with potassium peroxymonosulfate (PPMS), sodium hydroxide (NaOH), or acetic acid (HAc), showing no amplification signals. In simulated environmental samples (ASFV-spiked swabs from farm walls, rails, feed, floors, urine, and feces), the V-qPCR assay reliably detected inactivation in PPMS-treated samples (walls, rails, urine, feces) and NAOH-treated samples (walls, feces), showing no amplification. In contrast, HAc-inactivated samples yielded residual amplification (Ct ~ 31.5), suggesting superior performance of PPMS and NAOH in complex matrices. Validation using 30 ASFV-positive field environmental swabs revealed three outcomes: (i) 21 samples showed no amplification (confirmed inactivated); (ii) six samples showed similar Ct values before and after heat treatment (ΔCt [Ct_(+dye)_–Ct_(no dye)_] < 2.5, indicating inactivation); and (iii) three samples demonstrated detectable Ct values before heat inactivation but no amplification after treatment (confirming the presence of viable virus). This study represents the first application of V-qPCR for field-based assessment of ASFV infectivity, offering a rapid and practical tool to enhance diagnostic accuracy and biosecurity management in pig farms.

**Supplementary Information:**

The online version contains supplementary material available at 10.1186/s13567-025-01641-6.

## Introduction

African swine fever (ASF), caused by African swine fever virus (ASFV), is a highly contagious hemorrhagic disease affecting domestic pigs and wild boars. Characterized by high virulence, mortality rates approaching 100% in acute cases, and remarkable environmental persistence, ASF outbreaks have caused devastating socio-economic consequences worldwide [1]. The global spread of the disease has severely disrupted international pork trade, resulting in massive economic losses to the swine industry.

ASFV demonstrates complex transmission ecology involving multiple pathways within and between swine populations. Primary transmission routes include direct contact with infected animals (domestic pigs and wild boars) and biological vectors, particularly soft ticks of the *Ornithodoros* genus. Indirect transmission occurs through various fomites, including contaminated feed, water sources, pork products, semen, and farming equipment. Personnel movement and transport vehicles also serve as important mechanical vectors for viral dissemination [2]. Currently, no safe and effective vaccines or specific therapies are available for ASF; hence, biosecurity measures remain crucial for outbreak management. In affected countries and regions, detection, disinfection, and culling are primary control measures.

Traditionally, virus isolation has been the gold standard for assessing ASFV infectivity. However, it is time-consuming and requires biosafety level 3 (BSL-3) facilities, highlighting the urgent need for rapid and accurate detection technologies capable of discerning the infectivity status of samples. Nucleic acid-based detection methods, such as real-time quantitative PCR (qPCR), have been widely used for ASFV surveillance, offering high sensitivity and specificity. Nonetheless, following disinfection, viral genetic materials often remain largely intact [3]. Moreover, the multilayered architecture of ASFV shields the genome and helps maintain capsid stability [4]. The DNA genome of ASFV is highly resistant to degradation in the environment [5, 6].

**Figure 1:**
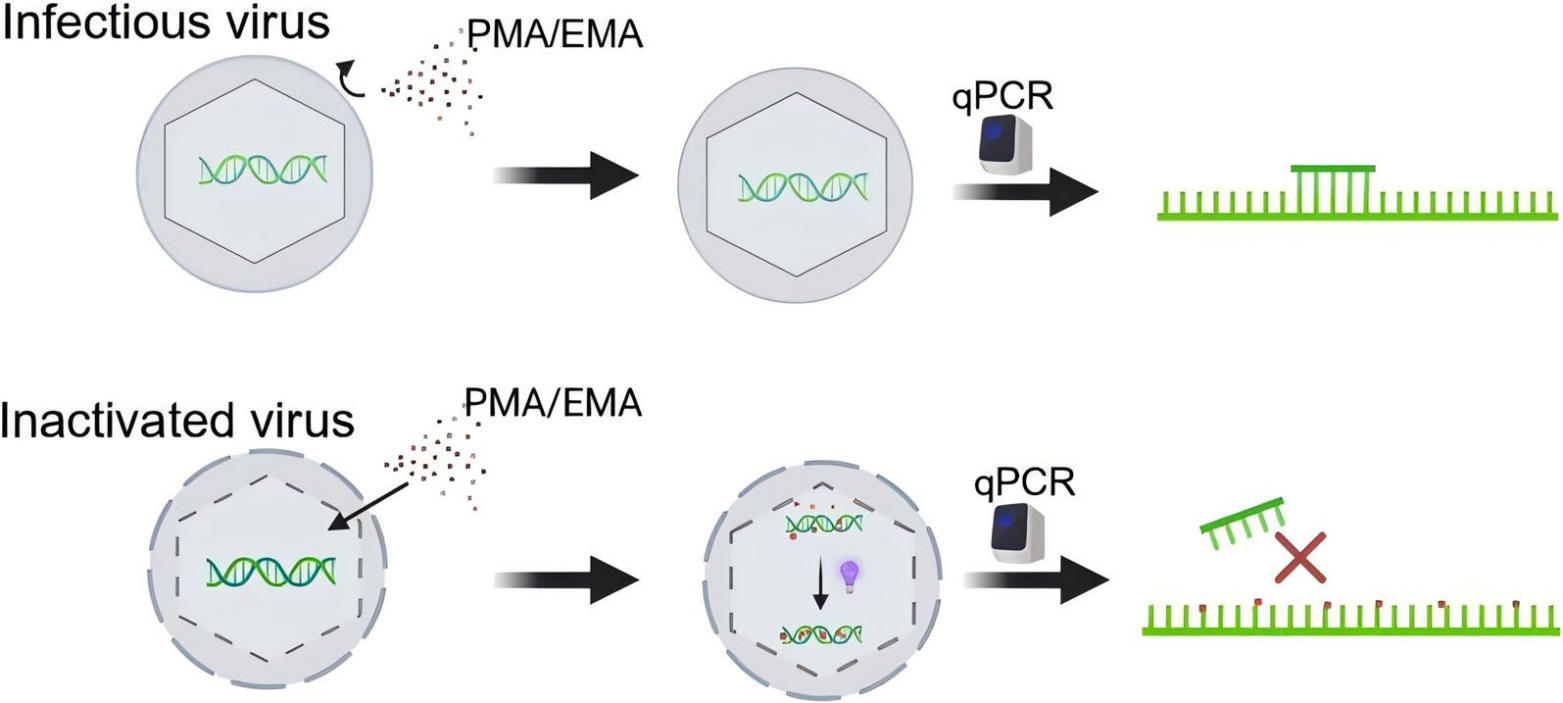
**Schematic diagram of V-qPCR for discriminating viable and inactivated ASFV.** PMA or EMA (represented as red dots) selectively penetrates damaged membranes of inactivated ASFV virions. Upon photoactivation, the dyes covalently cross-link with viral DNA, thereby inhibiting qPCR amplification. In contrast, the genomic DNA from viable ASFV remains accessible and is normally amplified.

Viability qPCR (V-qPCR), based on photosensitive nucleic acid dyes such as propidium monoazide (PMA) or ethidium monoazide (EMA), overcomes the limitations of traditional qPCR by differentiating viable and inactivated viruses or bacteria. In this approach, samples are pretreated with photosensitive nucleic acid dyes prior to DNA extraction. The dyes penetrate damaged or destroyed viral capsids but not intact ones. Following photoactivation, they intercalate covalently into nucleic acid chains, inhibiting PCR amplification (Figure 1). Over the past few decades, V-qPCR has been widely used to assess the infectivity of various enteric viruses, such as hepatitis A virus (HAV), hepatitis E virus (HEV), rotavirus (RV), adenovirus, norovirus, and Aichi virus (AiV), in environmental and food matrices [7−12]. It has also been applied in disease surveillance, environmental monitoring, and food and feed safety [13−15]. Recently, several studies have reported V-qPCR assays based on PMA or PMAxx combined with triton X-100 to distinguish viable and inactivated ASFV [16−18]. However, these experiments were conducted solely under laboratory conditions and did not adequately account for the complexity of sample matrices encountered in the field. They also neglected the potential application of EMA in scenarios where PMA was less effective.

In this study, PMA- and EMA-based V-qPCR assays were established and comprehensively evaluated to rapidly distinguish viable ASFV in environmental samples. This approach provides guidance for effective ASF control strategies and a potential tool for assessing disinfection efficacy in pig farms.

## Materials and methods

### Cells and virus strains

Porcine alveolar macrophages (PAMs) were cultured in RPMI 1640 medium (Gibco, China) supplemented with 10% fetal bovine sera (FBS) (Sigma, USA) and 1% antibiotics-antimycotics (10 000 IU/mL penicillin, 10 000 μg/mL streptomycin, and 25 μg/mL amphotericin B) at 37 °C in a CO_2_ incubator. The recombinant virus ASFV-ΔMGF-EGFP, a derivative of the ASFV HLJ/2018 strain (GenBank accession no. MK333180.1) with the *MGF* genes replaced by the enhanced green fluorescent protein (*EGFP*) gene, was propagated in PAMs as described previously [19]. Viral titers were determined using the Reed & Muench method [20]. All experiments involving live ASFV were performed in BSL-3 facilities at the Harbin Veterinary Research Institute (HVRI) of the Chinese Academy of Agricultural Science (CAAS). Pseudorabies virus (PRV) variant TJ strain [21] and atypical porcine pestivirus (APPV) China/HLJ491/2017 strain [22] were isolated and maintained in our laboratory. Classical swine fever virus (CSFV) attenuated live vaccine (C-strain), porcine circovirus 2 (PCV2) LG strain, and porcine reproductive and respiratory syndrome virus (PRRSV) HUN4-F112 strain were obtained from Harbin Weike Biotechnology Co., Ltd.

### Optimization of V-qPCR for assessing ASFV infectivity

The basic V-qPCR protocol was established as follows: 50 µL of PMA or EMA (20 µM; Biotium, USA) was mixed with 200 µL of heat-inactivated ASFV (105 °C for 60 min; 10^4.5^ TCID_50_/mL), and incubated in the dark at room temperature (26 °C) for 15 min, followed by photoactivation for 15 min using a PMA-Lite device (Biotium, USA). DNA was then extracted from each sample, and subjected to qPCR. To establish a sensitive and specific V-qPCR for effectively discriminating viable and inactivated ASFV, key parameters were optimized, including dye concentration, dark incubation time, and photolysis time, based on the above basic conditions [23−25]. Briefly, 50 µL of PMA or EMA (0, 5, 10, 20, 40, or 80 µM) was mixed with 200 µL of heat-inactivated ASFV (105 °C for 60 min; 10^4.5^ TCID_50_/mL). The mixtures were incubated in the dark at room temperature for 0, 10, 15, or 20 min, followed by photolysis for 0, 10, 15, or 20 min. Subsequently, qPCR was performed using the ASFV qPCR kit (NECVB, China) designed by our team.

To optimize the inhibitory effect of PMA or EMA on PCR amplification, we evaluated the potential synergistic effects of three commonly used surfactants, triton X-100 (Merck, USA), tween 20 (Solarbio, China) and sodium dodecyl sulfate (SDS; Amresco, USA). A mixture containing 50 µL of dye (20 μM) and 0.0001% to 0.01% triton X-100, tween 20, or SDS was added to 200 µL of heat-inactivated ASFV (105 °C for 60 min; 10^4.5^ TCID_50_/mL). Dye-treated PBS was used as a control. After photoactivation for 15 min, DNA was extracted and subjected to qPCR, with each sample tested in triplicate.

### Specificity test of V-qPCR

To determine the specificity of the V-qPCR assay, CSFV, PRRSV, PRV, PCV2, and APPV were tested using the V-qPCR designed for ASFV, with each sample run in triplicate.

### Sensitivity test of V-qPCR

Infectious ASFV (10^4.5^, 10^3.5^, 10^2.5^, 10^1.5^, and 10^0.5^ TCID_50_/mL) was examined by V-qPCR, with PBS used as a negative control. Each sample was tested in triplicate.

### Inactivation of ASFV by thermal or chemical treatments

ASFV stock solution (10^7.5^ TCID_50_/mL) was ten-fold serially diluted to 10^4.5^ TCID_50_/mL. The viral suspensions were then heat-treated for 60 min at 60 °C, 90 °C, or 105 °C. Finally, the samples were centrifuged at 12 000 × *g* (4 °C) for 2 min, and the supernatants were collected and stored at –80 °C until use.

For chemical inactivation, 180 μL of ASFV (10^4.5^ TCID_50_/mL) was mixed with 20 μL of one of the following: 10% (w/v) potassium peroxymonosulfate (PPMS; Virkon S, Lanxess, Germany), 10% (w/v) sodium hydroxide (NaOH; Sinopharm, China), 10% (v/v) acetic acid (HAc; Sinopharm, China), or distilled water (control). After incubation for 30 min at room temperature, the mixtures were immediately neutralized by adding 20 µL of an appropriate neutralizing agent: 10% (w/v) sodium thiosulfate (Na_2_S_2_O_3_; Bodi, China) for PPMS, 10% (w/v) HCl (Sinopharm, China) for NaOH, or 10% (w/w) NaOH for HAc. To assess infectivity after chemical treatment, PAMs (5 × 10^4.0^ cells per well) seeded in 96-well plates were incubated with the neutralized mixtures at a 1:1 ratio and maintained at 37 °C with 5% CO_2_. EGFP expression was monitored using fluorescence microscopy at 2 − 4 days post-inoculation. Additionally, a subset of chemically inactivated samples was tenfold diluted with PBS  without neutralization and subjected to the same infectivity assay.

### Preparation of simulated ASFV-positive environmental samples and thermal or chemical inactivation

Environmental samples from pig farms often contain complex matrices, making ASFV infectivity assessment by virus isolation challenging. To evaluate the potential of V-qPCR for differentiating viable and inactivated ASFV in the field, infectious ASFV was spiked into ASFV-negative environmental samples collected from pig farms.

Environmental samples (swabs from walls, rails, feed, floors, urine, and feces) were collected from clinically healthy pig farms and rinsed with PBS. After centrifugation at 12 000 × *g* for 2 min, the supernatants were collected and confirmed to be ASFV-negative using the ASFV qPCR kit designed by our team. These negative samples were then spiked with ASFV stock (10^7.5^ TCID_50_/mL) to reach a final titer of approximately 10^4.5^ TCID_50_/mL. Each mixture was divided into five aliquots. Four aliquots were inactivated by heat treatment (105 °C) or chemical disinfectants (PPMS, NaOH, or HAc) as described above, while one aliquot served as a non-inactivated control. All samples were then tenfold diluted with PBS. For the disinfectant-inactivated groups (PPMS, NaOH, or HAc), each suspension was subdivided and mixed at a 4:1 ratio with PBS, PMA, or EMA. For the non-inactivated and heat-inactivated groups, aliquots were treated with PBS, PMA or EMA at a 4:1 ratio, or with PMA + SDS, or EMA + SDS at a 4:1:0.1 ratio.

### Evaluation of V-qPCR for detecting clinical samples

Thirty environmental swabs from pig farms that tested qPCR-positive for ASFV were divided into two aliquots. The first aliquot was further divided into three parts. Each suspension (200 µL) was supplemented with 50 µL of PMA, EMA, or PBS. The second aliquot was treated at 105 °C for 60 min and then subjected to the same three treatments.

### Quantitative real-time PCR

Nucleic acids were extracted from all samples using the TGuide S32 Magnetic Viral DNA/RNA Kit (Tiangen, China). The qPCR reaction system (20 μL in total) consisted of 10 μL of 2 × reaction mix (Applied Biosystems, USA), 1 μL of forward primer (10 μM), 1 μL of reverse primer, 0.5 μL of probe (10 μM), 4.5 μL of DNase-free water, and 3 μL of template DNA. qPCR was performed on the QuantStudio 5 Real-time PCR System (Applied Biosystems, USA) with an initial denaturation step at 95 °C for 20 s, followed by 40 cycles of denaturation at 95 °C for 5 s and annealing/extension at 60 °C for 20 s.

### Statistical analysis

The ΔCt value was defined as the difference between the average Ct value of dye-treated samples and that of dye-untreated samples. All data were analyzed and graphically presented using GraphPad Prism version 6 (GraphPad Software, USA). Data are expressed as mean values ± standard deviations (SD). A *P*-value of less than 0.05 was considered statistically significant.

## Results

### Optimization of PMA and EMA treatments

**Figure 2:**
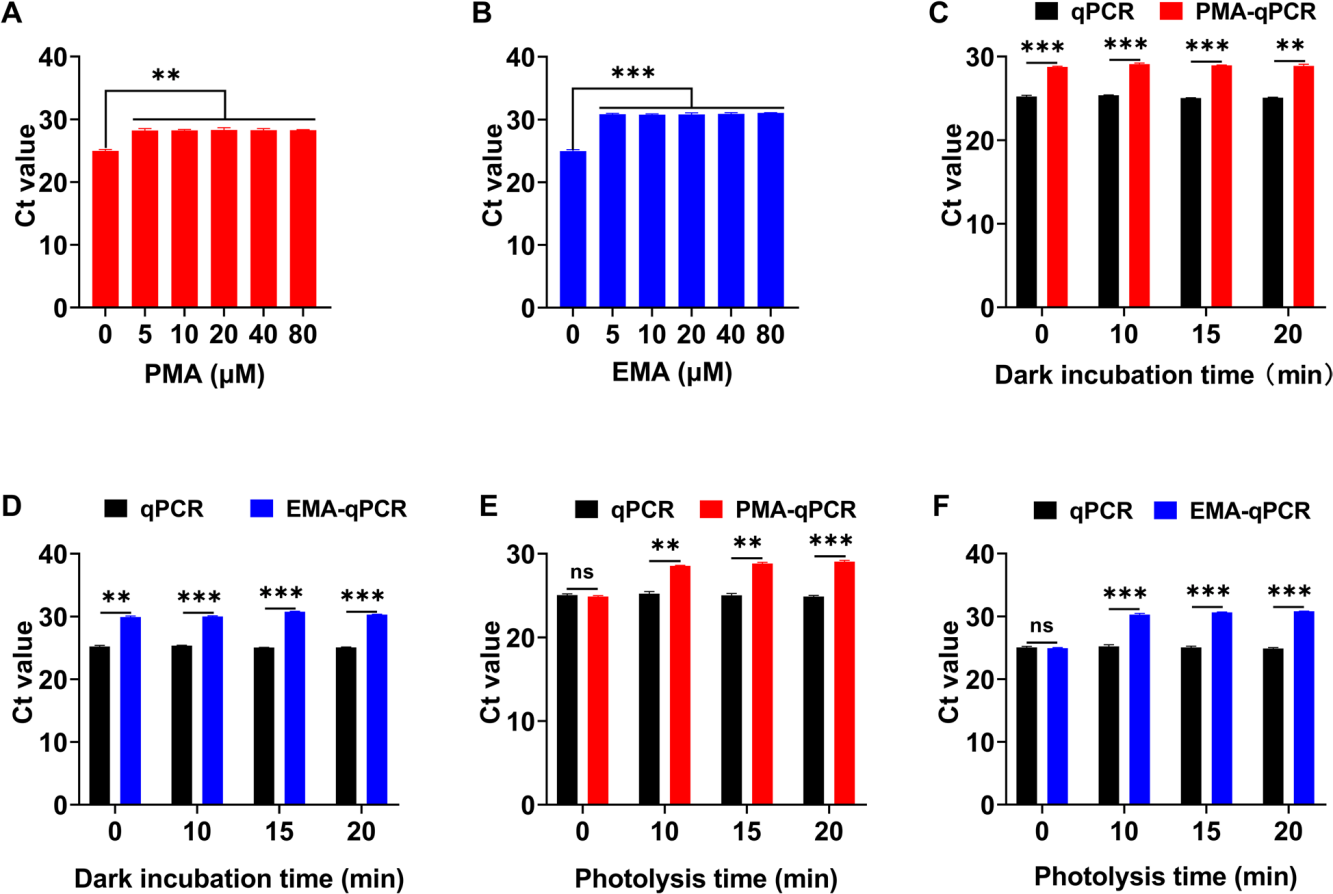
**Optimization of the working conditions of the V-qPCR assay for detecting heat-inactivated ASFV**. **A**, **B** Optimization of PMA and EMA dye concentrations. **C**, **D** Optimization of dark incubation time. **E**, **F** Optimization of photolysis time. *P < 0.05, **P < 0.01; ***P < 0.001; ns, not significant.

The V-qPCR assay was optimized, and the results showed no significant differences in Ct values under the following conditions: dye concentrations from 5 to 80 μM (Figures 2A and B), dark processing time from 0 to 20 min (Figures 2C and D), and photolysis time from 10 to 20 min (Figures 2E and F). Considering efficiency and cost, the dark incubation step was omitted, and 20 μM dye concentration and 15 min photolysis time were selected for further study.

### Effects of surfactants on the performance of V-qPCR for differentiating viable and inactivated ASFV

**Figure 3:**
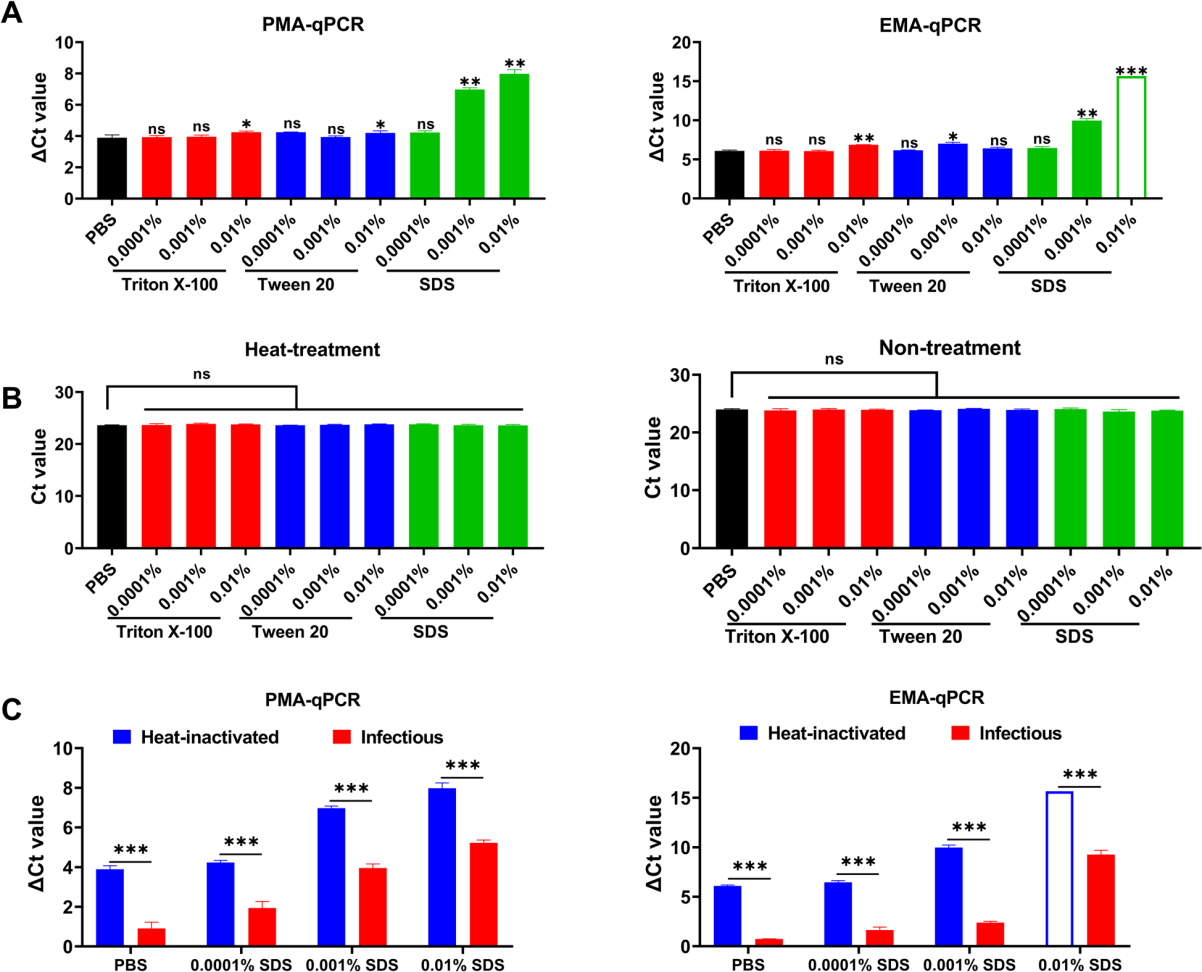
**Effects of surfactants on the performance of V-qPCR in discriminating viable and inactivated ASFV. A** V-qPCR detection of heat-inactivated ASFV (10^4.5^ TCID_50_/mL) in the presence of different concentrations (0.0001%, 0.001%, and 0.01%) of triton X-100, tween 20, or SDS. Dye-treated PBS without surfactant served as the control. **B** Effects of surfactants on qPCR detection of infectious and heat-inactivated ASFV in the absence of dye, with PBS as a control. **C** Effects of SDS on V-qPCR detection of viable and inactivated ASFV, with dye-treated PBS without SDS as a control. Unfilled columns represent no amplification and Ct values were set to 40 for ΔCt calculation. *P < 0.05, **P < 0.01; ***P < 0.001; ns, not significant.

The synergistic effects of three surfactants, triton X-100, tween 20, and SDS, on the V-qPCR performance were evaluated using 105 °C-inactivated ASFV (10^4.5^ TCID_50_/mL) . The addition of SDS significantly increased ΔCt values compared to dye-treated PBS controls. For the PMA-qPCR, Ct values increased by 4.2, 7.0, and 8.0 with 0.0001%, 0.001%, and 0.01% SDS, respectively. Corresponding increases for the EMA-qPCR were 6.5, 10.0, and 15.7. In contrast, neither triton X-100 nor tween 20 significantly affected the Ct values of V-qPCR (Figure 3A).

To exclude potential direct effects of surfactants on qPCR amplification, we assessed their impact on the detection of both viable and heat-inactivated ASFV in the absence of PMA or EMA. The results revealed no significant differences in Ct values between inactivated and infectious ASFV following surfactant treatment alone (Figure 3B). We also evaluated surfactant effects on dye-treated infectious ASFV. As shown in Figure 3C, the Ct values of dye-treated, heat-inactivated samples increased by 2.3, 3.0, and 2.8 for the PMA-qPCR with 0.0001%, 0.001%, and 0.01% SDS, respectively, compared to the infectious virus groups. For the EMA-qPCR, the increases were 4.8, 7.6, and > 6.4, respectively. To minimize the impact of surfactants on the detection of infectious virus while maximizing the ΔCt values between inactivated and infectious virus, a concentration of 0.001% SDS was selected for subsequent experiments.

### Specificity and sensitivity of V-qPCR

**Figure 4:**
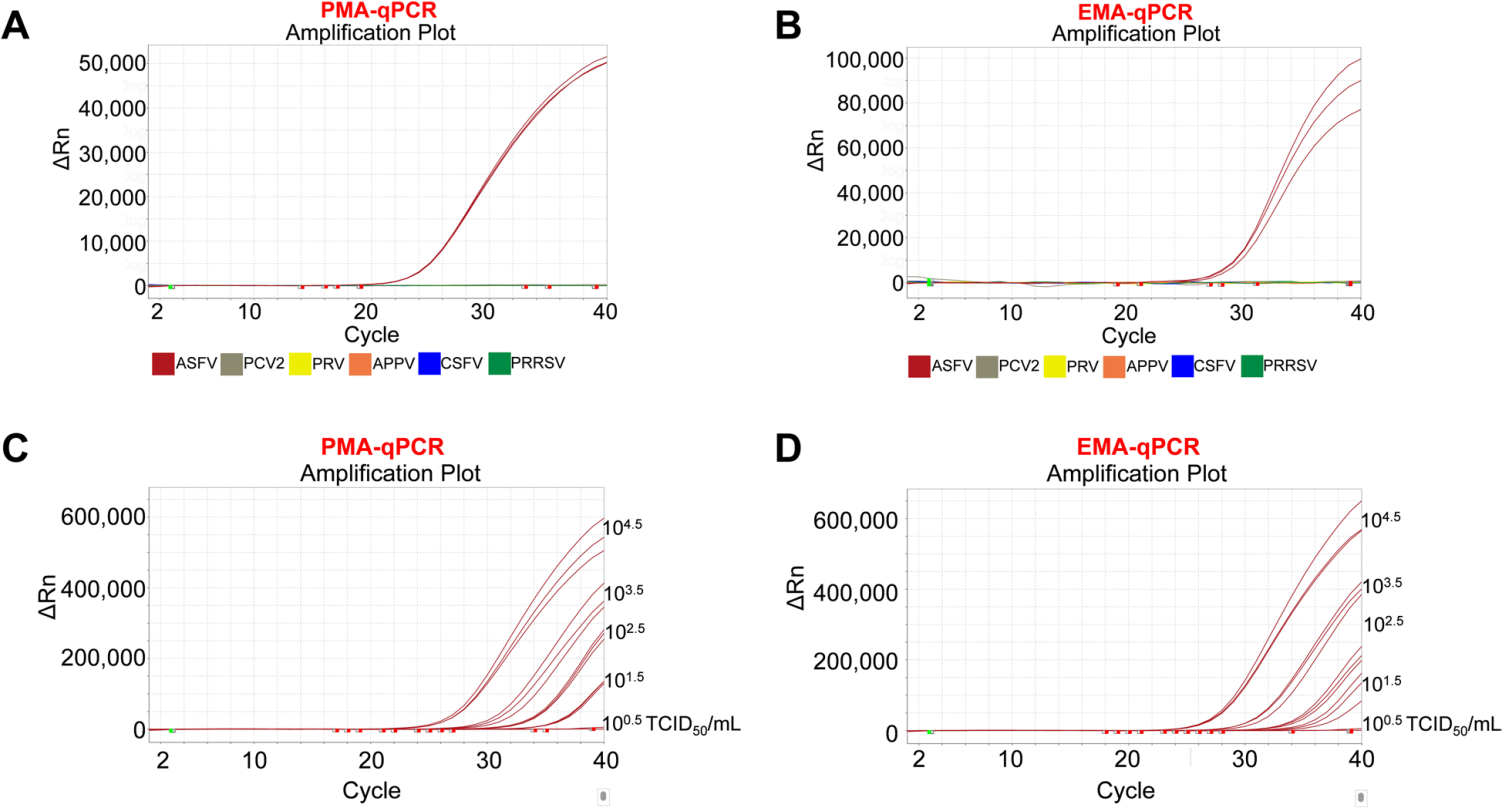
**Specificity and sensitivity of V-qPCR for detecting ASFV. A**, **B** Specificity testing of the V-qPCR assay against other common porcine viruses, including CSFV, PRRSV, PRV, PCV2, and APPV. **C**, **D** Sensitivity testing of the V-qPCR assay using serial dilutions of infectious ASFV (10^4.5^ to 10^0.5^ TCID_50_/mL). PBS was used as a negative control.

Specificity of the V-qPCR assay was evaluated by testing other common porcine viruses. As expected, this assay successfully amplified ASFV DNA and showed no cross-reaction with non-ASFV swine viruses, including CSFV, PRRSV, PRV, PCV2, and APPV (Figures 4A and B). Both PMA-qPCR and EMA-qPCR demonstrated a detection limit of 10^1.5^ TCID_50_/mL for ASFV (Figures 4C and D).

### Evaluation of the V-qPCR assay for detecting ASFV inactivated by thermal treatment and chemical disinfectants

**Figure 5:**
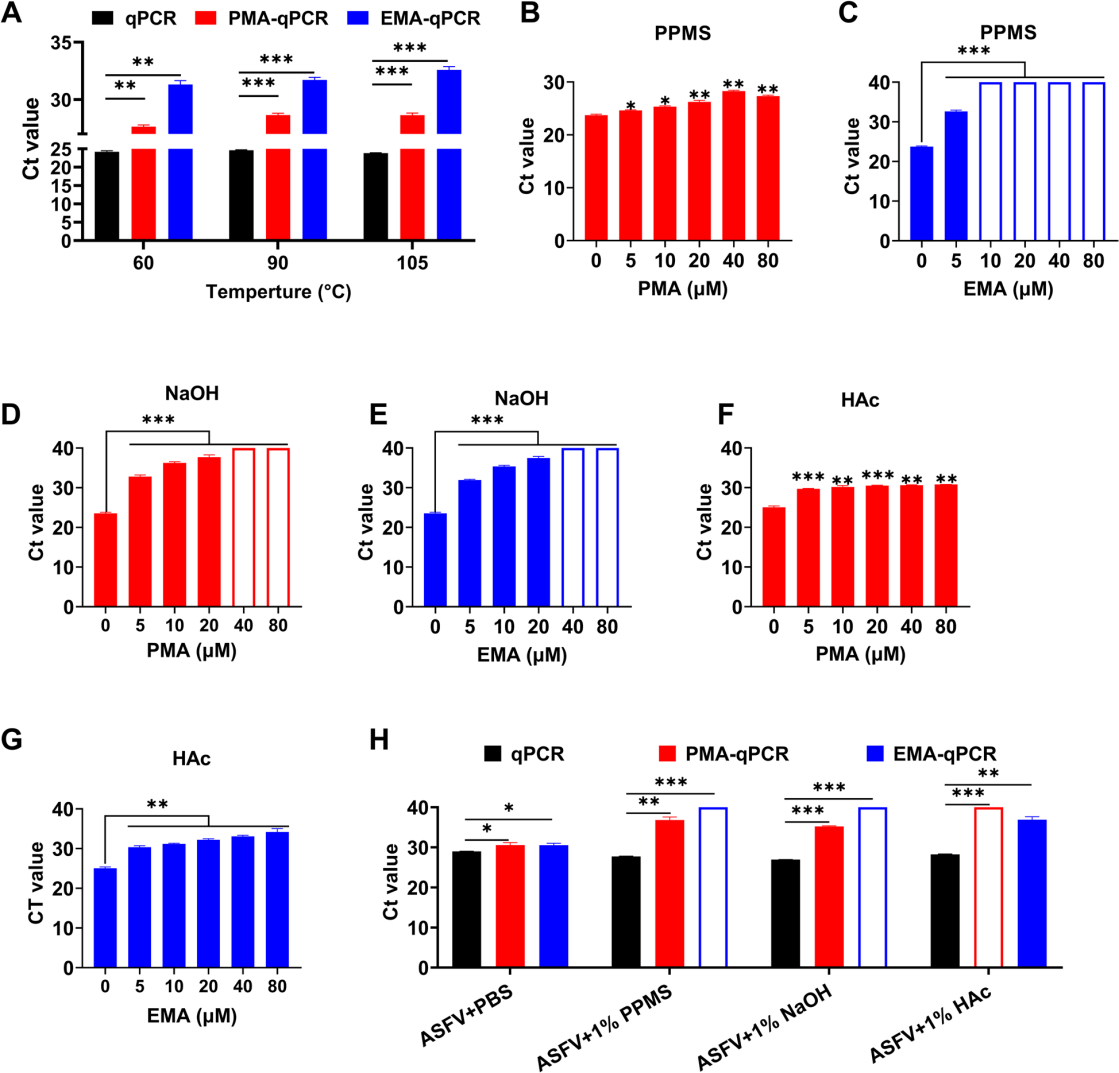
**Evaluation of V-qPCR for detecting ASFV inactivated by thermal treatment or chemical disinfectants. A** Comparison of qPCR and V-qPCR for detecting ASFV inactivated by heat treatment (60 °C, 90 °C, or 105 °C for 60 min; initial titer 10^4.5^ TCID_50_/mL). **B − G** Optimization of PMA and EMA dye concentrations for detecting ASFV inactivated by disinfectants PPMS (**B**, **C**), NaOH (**D**, **E**), or HAc (**F**, **G**). **H** Comparison of qPCR and V-qPCR for detecting ASFV inactivated by PPMS, NaOH, or HAc. After inactivation, samples were tenfold diluted with PBS prior to analysis. Data are presented as mean ± SD of three independent replicates. Unfilled columns indicate no amplification and Ct values were set to 40 for ΔCt calculation. *P < 0.05, **P < 0.01; ***P < 0.001; ns, not significant.

When detecting 60 °C, 90 °C, or heat-inactivated ASFV suspensions by V-qPCR, dye-treated samples showed significantly higher Ct values than untreated groups. The corresponding ΔCt values were 3.4, 4.0, and 4.8 for the PMA-qPCR, and 7.1, 7.0, and 8.8 for the EMA-qPCR (Figure 5A). These results indicate that high-temperature (105 °C) inactivation is more suitable for V-qPCR detection, and the EMA-qPCR outperforms the PMA-qPCR for heat-treated ASFV.

We further evaluated the assay using three disinfectants commonly used in pig farms, PPMS, NaOH, and HAc. As shown in Additional file 1, fluorescence signals were observed only in the distilled water control group, confirming sufficient viral inactivation by all disinfectants. To account for potential effects of disinfectants on sample conditions (e.g., pH and ion concentration), dye concentrations were re-optimized for detecting chemically inactivated ASFV. The optimal dye concentrations were determined to be 40 μM (PMA) and 10 μM (EMA) for PPMS-inactivated ASFV, 40 μM for NaOH-inactivated ASFV, and 20 μM for HAc-inactivated ASFV (Figures 5B−G).

Notably, when disinfectant-inactivated ASFV suspensions were tenfold diluted with PBS and detected by V-qPCR, the PMA-qPCR yielded significantly increased Ct values of 36.8, 35.2, or no amplification (ΔCt values of 9.1, 8.3, or 11.7, respectively) compared with dye-untreated samples. In contrast, the EMA-qPCR produced no detectable amplification signals for PPMS- and NaOH-inactivated samples (ΔCt values of 12.3 and 13.1, respectively), while a Ct value of 36.9 (ΔCt = 10.0) was observed for HAc-inactivated samples (Figure 5H). These results suggest that the EMA-qPCR assay is more suitable for assessing PPMS- and NaOH-inactivated ASFV, whereas the PMA-qPCR assay performs more effectively for HAc-inactivated virus. Notably, for disinfectant-inactivated samples, no DNA amplification was observed by either PMA- or EMA-qPCR even in the absence of SDS, indicating that surfactants are unnecessary for chemically inactivated samples.

### Performance of V-qPCR for differentiating viable and inactivated ASFV in the field

**Figure 6:**
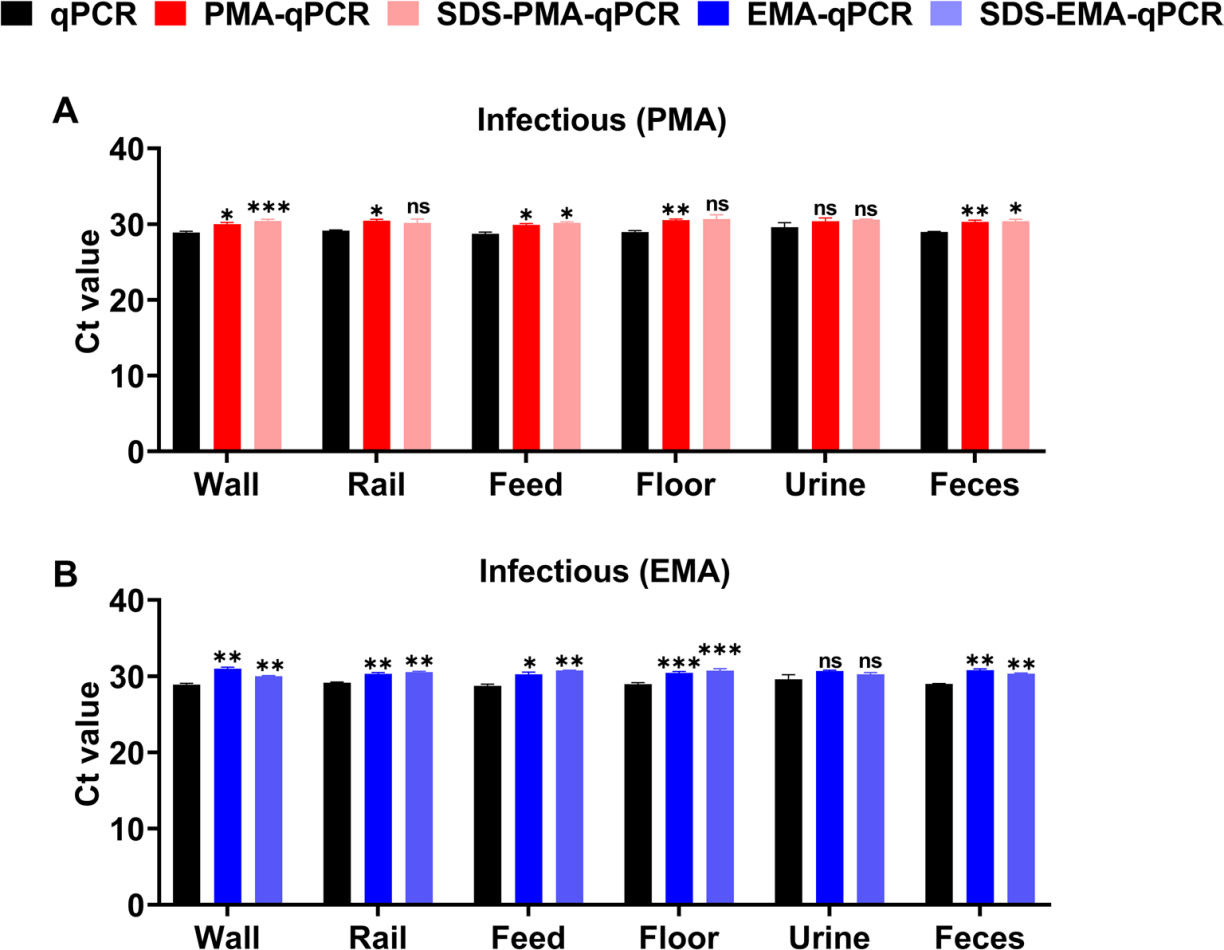
**Evaluation of the effects of environmental sample matrices on viable ASFV detection using V-qPCR. A**, **B** Infectious ASFV was spiked into negative environmental samples (swabs from walls, rails, feed, floors, urine, and feces) collected from pig farms to a final titer of 10^4.5^ TCID_50_/mL. Samples were then tenfold diluted with PBS and detected by qPCR and V-qPCR. *P < 0.05, **P < 0.01; ***P < 0.001; ns, not significant.

To evaluate the potential of V-qPCR for differentiating viable and inactivated ASFV in the field, infectious ASFV was added to negative environmental samples (swabs from walls, rails, feed, floors, urine, and feces) collected from pig farms to create simulated ASFV-positive environmental samples. When detecting simulated viable (non-inactivated) environmental samples by the V-qPCR, ΔCt values were less than 2.5, regardless of SDS addition (Figures 6A and B).

**Figure 7:**
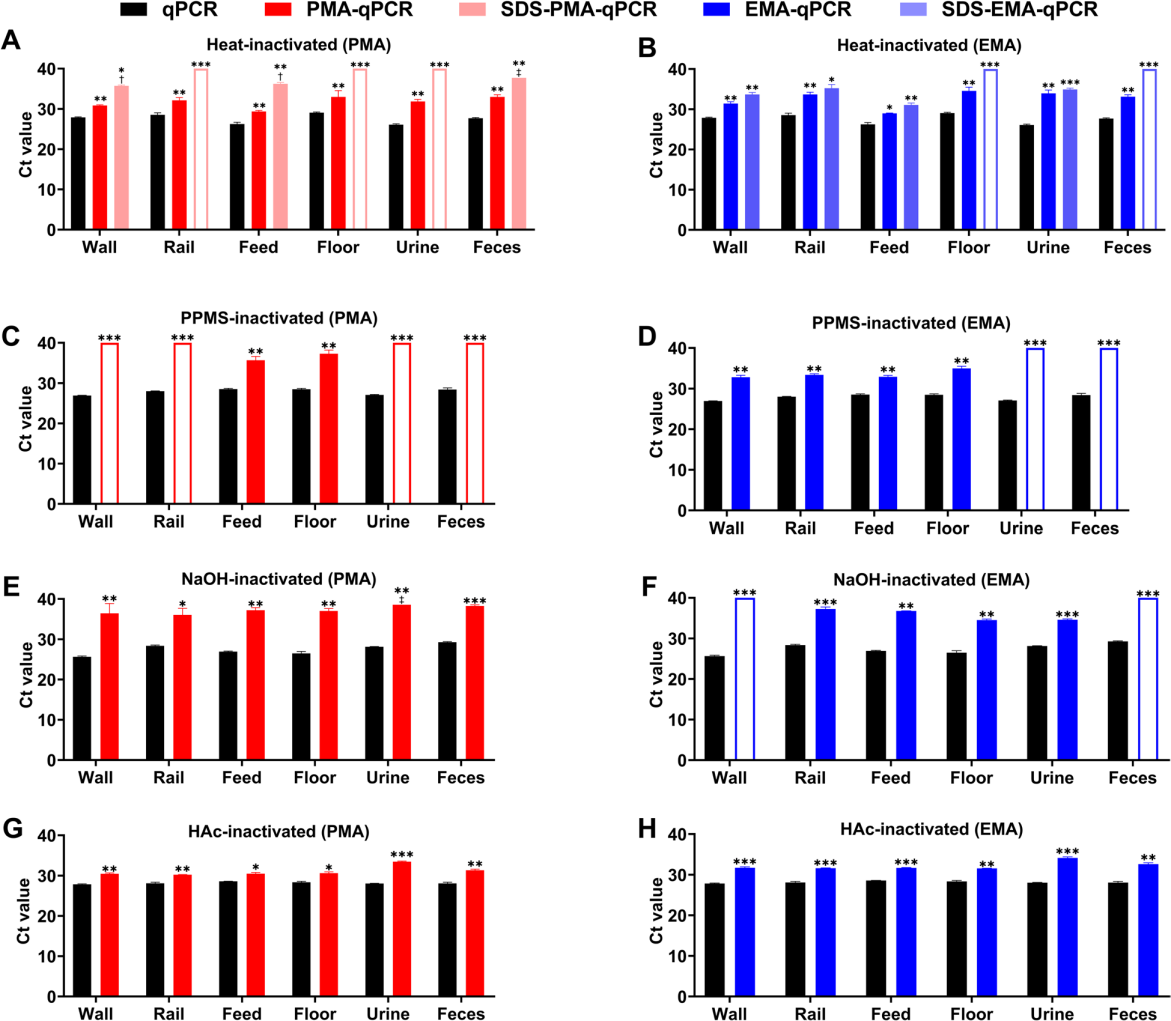
**Performance of V-qPCR for differentiating viable and inactivated ASFV in simulated environmental samples. A − H** Detection of simulated environmental samples inactivated by 105 °C (**A**, **B**), PPMS (**C**, **D**), NaOH (**E**, **F**), or HAc (**G**, **H**) using qPCR and V-qPCR. Infectious ASFV was spiked into negative environmental swabs from walls, rails, feed, floors, urine, and feces to a final titer of 10^4.5^ TCID_50_/mL, and inactivated. Samples were then tenfold diluted with PBS before analysis. Unfilled columns represent no amplification and Ct values were set to 40 for ΔCt calculation. †, one of the three repeats with no amplification. ‡, two of the three repeats with no amplification. *P < 0.05, **P < 0.01; ***P < 0.001; ns, not significant.

For simulated samples inactivated at 105 °C, Ct values increased significantly (*P* < 0.001) compared to dye-free controls for both PMA-qPCR and EMA-qPCR, with ΔCt values ranging from 3.0 to 5.8 and 2.8 to 7.8, respectively. The addition of SDS further increased ΔCt values to 7.9–13.9 and 4.9–12.3, respectively. Notably, no amplification was observed in rail, floor, and urine samples by the PMA-qPCR (ΔCt 10.9–13.9) and in floor and feces samples by EMA-qPCR (ΔCt 10.9 and 12.3) (Figures 7A and B). These results indicate that SDS improves V-qPCR performance for detecting heat-inactivated samples in complex matrices. For PPMS-inactivated samples, no amplification was observed in wall, rail, urine, and feces samples when detecting by PMA-qPCR (ΔCt 11.6–13.1), while ΔCt values for feed and floor samples were 7.2 and 8.8, respectively (Figure 7C). No DNA amplification was observed in urine and feces samples (ΔCt 11.6–13.0) by the EMA-qPCR, while ΔCt values for other samples ranged from 4.4 to 6.5 (Figure 7D). For NaOH-inactivated samples, ΔCt values were 7.7–10.8 by PMA-qPCR (Figure 7E), while no amplification was achieved in wall and feces samples by EMA-qPCR, (ΔCt 14.3 and 10.7), with other samples showing ΔCt values from 6.4 to 9.8 (Figure 7F). HAc-inactivated samples exhibited the lowest performance, with ΔCt values ranging from approximately 1.9 to 6.1 (Figures 7G and H). Collectively, these data suggest that the V-qPCR assay is well-suited for monitoring environmental samples inactivated by high temperature (105 °C), PPMS and NAOH in the field.

Subsequently, 30 ASFV PCR-positive environmental swabs were analyzed by both the PMA- and the EMA-qPCR. Of these, 21 samples showed no DNA amplification. Six samples exhibited similar Ct values (difference < 2.5) before and after 105 °C treatment, indicating inactivation. Three samples (No. 2, 13, and 22) showed Ct values prior to heatinactivation but no DNA amplification after treatment, confirming the presence of viable ASFV (Table 1).

**Table 1:** **Detection of ASFV in field environmental samples from pig farms using V-qPCR**.

Sample No.	Untreated			Heat-inactivated			Results
	Ct_(no dye)_	Ct_(+PMA)_	Ct_(+EMA)_	Ct_(no dye)_	Ct_(+PMA)_	Ct_(+EMA)_	
1	29.72 ± 0.22	NA	NA	29.83 ± 0.30	NA	NA	–
2	30.40 ± 0.25	33.27 ± 0.10	33.67 ± 0.50	30.31 ± 0.31	NA	NA	+
3	30.16 ± 0.08	NA	NA	30.25 ± 0.09	NA	NA	–
4	30.34 ± 0.11	NA	NA	32.70 ± 0.23	NA	NA	–
5	29.76 ± 0.34	NA	NA	29.81 ± 0.07	NA	NA	–
6	30.34 ± 0.12	NA	NA	32.70 ± 0.24	NA	NA	–
7	29.78 ± 0.34	NA	NA	29.92 ± 0.08	NA	NA	–
8	29.77 ± 0.35	NA	NA	30.65 ± 0.19	NA	NA	–
9	30.95 ± 0.50	NA	NA	31.49 ± 0.11	NA	NA	–
10	32.87 ± 0.12	NA	NA	34.98 ± 0.76	NA	NA	–
11	30.07 ± 0.08	NA	NA	30.77 ± 0.42	NA	NA	–
12	35.60 ± 0.51	NA	NA	36.21 ± 0.73	NA	NA	–
13	28.83 ± 0.20	34.58 ± 0.08	34.67 ± 0.20	28.86 ± 0.08	NA	NA	+
14	31.21 ± 0.10	NA	35.67 ± 0.40	31.57 ± 0.06	NA	38.15 ± 0.25	–
15	32.27 ± 0.09	NA	NA	32.32 ± 0.09	NA	NA	–
16	32.41 ± 0.17	NA	NA	32.49 ± 0.24	NA	NA	–
17	36.69 ± 0.91	NA	NA	35.27 ± 0.58	NA	NA	–
18	28.93 ± 0.06	34.60 ± 0.37	34.93 ± 0.57	29.87 ± 0.19	36.44 ± 0.71	36.03 ± 0.67	–
19	32.13 ± 0.16	NA	NA	32.62 ± 0.13	NA	NA	–
20	31.37 ± 0.15	NA	NA	31.37 ± 0.10	NA	NA	–
21	28.91 ± 0.17	34.36 ± 0.17	33.77 ± 0.49	29.80 ± 0.12	34.68 ± 0.13	34.47 ± 0.55	–
22	30.72 ± 1.36	33.28 ± 0.15	35.25 ± 0.29	31.79 ± 1.92	NA	NA	+
23	32.92 ± 0.41	NA	NA	34.69 ± 1.82	NA	NA	–
24	33.26 ± 0.28	NA	NA	34.59 ± 1.95	NA	NA	–
25	32.96 ± 0.63	NA	NA	33.10 ± 0.66	NA	NA	–
26	32.78 ± 0.58	38.30 ± 0.50	NA	32.82 ± 1.54	NA	NA	–
27	30.28 ± 0.68	35.96 ± 0.85	NA	30.70 ± 0.42	35.97 ± 1.19^†^	NA	–
28	32.97 ± 1.93	NA	NA	32.29 ± 1.99	NA	NA	–
29	30.12 ± 0.44	NA	NA	31.11 ± 0.56	NA	NA	–
30	28.48 ± 1.10	NA	36.33 ± 1.09	30.93 ± 0.46	NA	36.73 ± 0.21^†^	–

NA, no amplification.

^†^one of three replicates showed no amplification.

+, infectious (samples showing detectable Ct values before 105 °C-inactivation but no amplification after inactivation).

–, inactivated (samples showing no significant change in Ct values (ΔCt < 2.5) or no amplification both before and after heat inactivation).

## Discussion

As the world's largest pork producer and consumer, China has suffered substantial economic losses since the initial ASF outbreak in 2018. ASFV is highly persistent in natural environments [26], making stringent disinfection and regular monitoring of disinfection efficacy critical for disease control and eradication. However, virus isolation, the gold standard for infectivity detection, is impractical for routine monitoring of infectious ASFV in field environments and for post-disinfection validation.

V-qPCR, which utilizes photosensitive nucleic acid dyes, offers several advantages over traditional virus isolation. It does not require BSL-3 facilities, making it suitable for resource-limited setting such as pig farms. Considering the significant time and labor investment associated with virus isolation, integrating V-qPCR into routine diagnostics provides a simpler and faster alternative for detecting viable ASFV on-site.

Despite its benefits, V-qPCR may encounter limitations, particularly with complex environmental samples. Matrices from pig farms may interfere with dye penetration and efficacy [27−30]. Previous studies have explored PMA-qPCR [16] and triton X-100-assisted PMAxx-qPCR [17] for distinguishing viable ASFV. However, these assays were conducted under controlled laboratory conditions and did not fully account for the complex matrices encountered in farm environments. They also overlooked the potential of EMA as an alternative dye where PMA may underperform. Balestreri et al. demonstrated the remarkable thermal stability of ASFV, noting that viable virus could still be detected by V-qPCR even after exposure to 100 °C for 20 min [18]. This finding highlights the necessity of robustly optimized V-qPCR protocols for accurately assessing viral viability under harsh conditions. In this study, we systematically optimized key parameters of the V-qPCR assay, including dye concentration, dark incubation time, and photoactivation duration [31, 32]. We also investigated the potential synergistic effects of surfactants, triton X-100, tween 20 and SDS, on the V-qPCR-based assessment of ASFV infectivity. Consistent with Hong et al., who reported an SDS-PMA-assisted RT-qPCR for rapid determination of viable SARS-CoV-2 [33], we identified SDS as the most effective surfactant for detecting heat-inactivated ASFV. This finding contrasts with Liu et al., who reported that triton X-100 alone improved the performance of PMAxx-qPCR for ASFV detection [17].

Applying the optimized V-qPCR assay to ASFV inactivated at 60 °C, 90 °C, and 105 °C revealed that samples treated at 105 °C yielded the highest ΔCt values (Ct_(+dye)_–Ct_(no dye)_). This indicates that high-temperature inactivation is most suitable for V-qPCR analysis. Furthermore, the EMA-qPCR demonstrated superior performance compared to the PMA-qPCR, suggesting that EMA is the preferred dye for detecting heat-inactivated ASFV.

To develop a V-qPCR applicable in the field, we spiked infectious ASFV into negative environmental samples (swabs from walls, rails, feed, floors, urine, and feces) collected from pig farms to simulate field conditions, as these samples represent important vectors for ASFV transmission. The results indicated that V-qPCR is suitable for testing samples inactivated by commonly used disinfectants like PPMS and NAOH, even in complex matrices. Furthermore, when we applied the V-qPCR assay to evaluate 30 ASFV PCR-positive environmental swabs from pig farms, 27 samples were considered inactivated, evidenced by no DNA amplification (21 samples) or similar Ct values before and after 105 °C inactivation (six samples), while three samples were confirmed to contain viable virus, showing detectable Ct values before heat inactivation and no amplification afterward. These data demonstrate the practical applicability of the V-qPCR assay in field settings. To our knowledge, this is the first report applying V-qPCR to evaluate ASFV infectivity in the field, offering a promising tool for assessing the efficacy of routine disinfection strategies in pig farms.

In summary, we established a V-qPCR assay capable of distinguishing viable and inactivated ASFV in both controlled and complex environmental conditions. The assay is particularly suitable for monitoring samples inactivated by high temperature (105 °C), PPMS, or NAOH in the field. Given the variety of disinfection strategies and the complexity of environmental matrices on pig farms, the concurrent application of both the PMA-qPCR and the EMA-qPCR assays is recommended for comprehensive clinical and field diagnostics.

## Supplementary Information

**Additional file 1. Verification of ASFV infectivity after chemical inactivation by inoculation of PAMs.** PAMs (5 × 10^4.0^ cells per well) in 96-well plates were inoculated with ASFV after chemical inactivation by PPMS, NaOH, or HAc at a 1:1 ratio and incubated at 37 °C with 5% CO_2_. EGFP expression was observed by fluorescence microscopy at 2 − 4 days post-inoculation, and representative fluorescence images were captured.

**Additional file 2.** **Official approval certificate for the ASFV quantitative PCR kit issued by the Ministry of Agriculture and Rural Affairs of China (Announcement No. 409).**

## Data Availability

The datasets generated and/or analyzed during the current study are available from the corresponding authors upon reasonable request. The data supporting the conclusions of this article are included within the article and its supplementary file. The ASFV qPCR detection kit (NECVB, China) referenced in this study was developed by our research team and has been officially approved by the Ministry of Agriculture and Rural Affairs of China (Additional file 2). Due to commercial confidentiality and intellectual property protection, the primer and probe sequences are proprietary and cannot be publicly disclosed. Researchers interested in collaboration or use of the kit for non-commercial purposes may contact the corresponding author for further information.
