# Supplementary material for: Rapid differentiation of viable and inactivated African swine fever virus by a viability quantitative PCR
Source: Vet Res. 2025 Oct 28;56:204. doi: 10.1186/s13567-025-01641-6 (PMC12570408; doi:10.1186/s13567-025-01641-6)
Supplement: Supplementary file 2 — Additional file 2. Official approval certificate for the ASFV quantitative PCR kit issued by the Ministry of Agriculture and Rural Affairs of China (Announcement No. 409). [file 13567_2025_1641_MOESM2_ESM.pdf]

中华人民共和国农业农村部公告

第409号

根据《兽药管理条例》和《兽药注册办法》规定，经审查，批准中国农业科学院哈尔滨兽医研究所等5家单位申报的非洲猪瘟病毒荧光PCR检测试剂盒产品注册，并发布产品试行规程、质量标准、说明书和内包装标签。自发布之日起执行。

特此公告。

附件：1. 注册目录

2. 制造及检验试行规程

3. 质量标准

4. 说明书和内包装标签

中华人民共和国农业农村部

农业农村部

2021年4月9日

附件 1

注册目录

产品名称	研制单位	备注
非洲猪瘟病毒荧光PCR检测试剂盒	中国农业科学院哈尔滨兽医研究所、哈尔滨国生生物科技股份有限公司、北京亿森宝生物科技有限公司、上海快灵生物科技有限公司、哈尔滨维科生物技术有限公司	注册
